# Provocative MRI catheterization

**DOI:** 10.1186/1532-429X-16-S1-P143

**Published:** 2014-01-16

**Authors:** Toby Rogers, Kanishka Ratnayaka, Jonathan R Mazal, William Schenke, Laurie Grant, Annette Stine, Peter Kellman, Anthony Z Faranesh, Robert J Lederman

**Affiliations:** 1 National Heart Lung and Blood Institute, National Institutes of Health, Bethesda, Maryland, USA

## Background

Invasive right heart catheterization plays a central role in the investigation of patients with cardiac and pulmonary vascular disease. Physiological provocations during invasive heart catheterization augment the diagnostic yield and can provide useful prognostic information. MRI catheterization combines invasive hemodynamic measurements with MRI structural and functional evaluations - thus providing superior diagnostic information than either test alone. An additional benefit to both patient and operator is no ionizing radiation, which is of particular value in pediatric patients.

## Methods

We propose a diagnostic algorithm for MRI catheterization integrating serial physiological provocations designed to unmask latent pathology and aid prognostication. The algorithm allows each MRI catheterization to be tailored to the individual patient. We have developed a concise MRI examination protocol to be performed alongside invasive hemodynamic measurements during each provocation. This protocol provides cardiac chamber volumes, pulmonary and systemic blood flow measurements with each provocation. The MRI examination is performed free breathing, which allows the study to be performed in patients with dyspnea or under moderate sedation.

## Results

**Figure 1:**
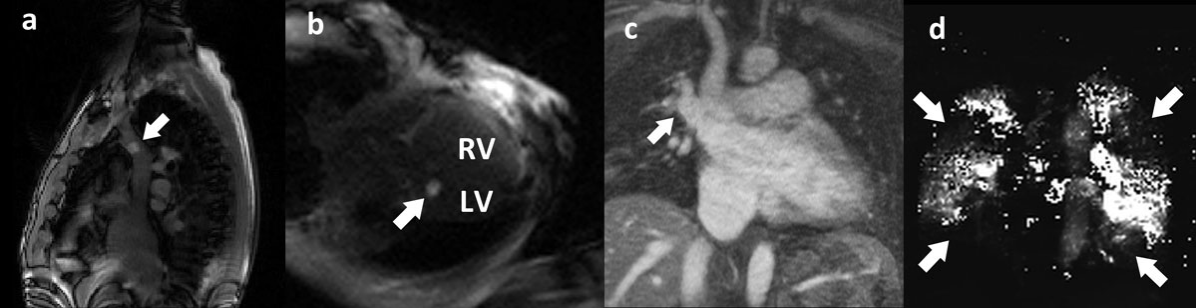
**MRI catheterization images (a) Gadolinium-filled balloon-tip (arrow) catheter in superior vena cava**. (b) Gadolinium-filled balloon-tip (arrow) catheter in left ventricle through atrial septal defect. (c) Anomalous pulmonary vein (arrow) draining into superior vena cava. (d) Lung perfusion map showing multiple filling defects (arrows).

Real-time MRI provides excellent anatomical imaging for catheter navigation. Challenging procedural steps using X-ray guidance, such as navigating into the SVC (Figure 1a) or left pulmonary artery, or crossing an atrial septal defect (Figure 1b), are easy using MRI-guidance and gadolinium-filled balloon-tip catheters. Physiological provocation can unmask pathology not apparent at rest. For example, patient A (Table 1) had normal cardiac chamber dimensions and pressures at rest but was breathless on exertion. With intravenous volume challenge, the left atrial volume, pulmonary capillary wedge pressure and pulmonary arterial pressure increased. The diagnosis is diastolic heart failure. Patient B had a severely dilated right ventricle. MRI catheterization identified an anomalous pulmonary vein (Figure 1c), which was easily engaged with the catheter from which oxygen saturations confirmed left-to-right shunt. Inhalation of nitric oxide resulted in 38% reduction in pulmonary artery pressure. The diagnosis is intra-cardiac shunt from anomalous pulmonary vein. Patient C had a history of deep vein thrombosis. MRI catheterization demonstrated dilated right ventricle with preserved systolic function and severe pulmonary arterial hypertension. MRI lung perfusion confirmed multiple defects throughout both lung fields (Figure 1d). Inhalation of nitric oxide resulted in a 25% reduction in pulmonary vascular resistance. The diagnosis is chronic thrombo-embolic pulmonary hypertension.

**Table 1:** Sample patient MRI catheterization data.

Patient	Physiological provocation	Cardiac chamber dimensions	Intracardiac pressures	Pulmonary vascular resistance	Qp:Qs	Diagnosis
**A**	Baseline (room air)	Mildly dilated left atrium	Normal pulmonary artery and wedge	Normal		**Diastolic LV dysfunction**

	10 mL/kg saline challenge	15% increase	55% increase	15% increase		

**B**	Baseline (room air)	Severely dilated right ventricle	Normal	Normal	2:1	**Anomalous pulmonary vein**

	100% O_2 _+ 40 ppm NO		38% reduction	68% reduction		

**C**	Baseline (room air)	Severely dilated right ventricle	Elevated pulmonary artery	Elevated		**Chronic thromboembolic pulmonary hypertension**

	100% O_2 _+ 40 ppm NO		17% reduction	25% reduction		

Only salient positive and negative findings are presented for clarity.

## Conclusions

Provocative MRI catheterization can uncover diagnoses not apparent at rest and can provide incremental structural and functional information compared with invasive heart catheterization or MRI alone.

## Funding

This work is supported by the Division of Intramural Research (Z01-HL005062-08, Z01-HL006039-01), National Heart Lung and Blood Institute, National Institutes of Health, USA.

